# The anticoagulation one year after ablation of atrial fibrillation in patients with atrial fibrillation (ALONE-AF) trial: Study protocol

**DOI:** 10.1016/j.heliyon.2024.e36506

**Published:** 2024-08-16

**Authors:** Daehoon Kim, Jaemin Shim, Eue-Keun Choi, Il-Young Oh, Jun Kim, Young Soo Lee, Junbeom Park, Jum-Suk Ko, Kyoung-Min Park, Jung-Hoon Sung, Hyung Wook Park, Hyung-Seob Park, Jong-Youn Kim, Hee Tae Yu, Tae-Hoon Kim, Boyoung Joung

**Affiliations:** a Division of Cardiology, Department of Internal Medicine, Yonsei University College of Medicine, Seoul, Republic of Korea; b Department of Cardiology, Korea University Hospital, Seoul, Republic of Korea; c Department of Cardiology, Seoul National University Hospital, Seoul, Republic of Korea; d Department of Cardiology, Seoul National University Bundang Hospital, Seongnam, Republic of Korea; e Heart Institute, University of Ulsan College of Medicine, Asan Medical Center, Seoul, Republic of Korea; f Division of Cardiology, Daegu Catholic University Hospital, Daegu, Republic of Korea; g Department of Cardiology, Ewha Womans University Hospital, Seoul, Republic of Korea; h Division of Cardiology, Department of Internal Medicine, Wonkwang University School of Medicine and Hospital, Iksan, Republic of Korea; i Division of Cardiology, Department of Medicine, Samsung Medical Center, Sungkyunkwan University School of Medicine, Seoul, Republic of Korea; j Division of Cardiology, CHA Bundang Medical Center, CHA University, Seongnam, Republic of Korea; k Division of Cardiovascular Medicine, Department of Internal Medicine, Chonnam National University Hospital, Gwangju, Republic of Korea; l Division of Cardiology, Keimyung University Hospital, Daegu, Republic of Korea; m Division of Cardiology, Department of Internal Medicine, Gangnam Severance Hospital, Seoul, Republic of Korea

**Keywords:** Atrial fibrillation, Catheter ablation, Anticoagulation, Study protocol

## Abstract

**Background:**

The ideal long-term antithrombotic strategy for patients after successful catheter-based atrial fibrillation (AF) ablation is still uncertain. Presently, practices vary, and the advantages of oral anticoagulation (OAC) for the post-ablation population are not clearly established. To date, no randomized trials have addressed this therapeutic question. This study aimed to evaluate whether no OAC therapy is superior to apixaban in reducing the risk of stroke, systemic embolism, or major bleeding among patients without apparent recurrent atrial arrhythmias for at least 1 year after their AF ablation procedure.

**Methods:**

The ALONE-AF trial is a prospective, multicenter, open-label, randomized study with blinded outcome assessment. Patients with AF who have at least one non-gender stroke risk factor (as determined by the CHA_2_DS_2_-VASc score) and no documented recurrences of atrial arrhythmia for at least 12 months post-ablation will be randomly assigned to apixaban 5 mg b.i.d. or no OAC therapy. The primary endpoint is a composite outcome of stroke, systemic embolism, and major bleeding. Key secondary outcomes include clinically relevant non-major bleeding, all-cause mortality, myocardial infarction, transient ischemic attack, quality of life, and frailty analysis. Participants will be followed for a period of 2 years. The estimated total sample size is 840 subjects, with 420 subjects in each arm.

**Conclusion:**

The ALONE-AF trial aims to provide robust evidence for the optimal anticoagulation strategy for patients with stroke risk factors following successful AF ablation.

**Clinical Trial Registration**: NCT04432220 (https://www.clinicaltrials.gov)

## 1 Background

Atrial fibrillation (AF) stands as the most prevalent form of sustained cardiac arrhythmia, imposing substantial economic and public health burdens [[1], [2], [3], [4]]. Compared to pharmacological therapy, AF catheter ablation (AFCA) has demonstrated efficacy in reducing acute episodes and prolonging periods of sinus rhythm, thereby enhancing patients' quality of life [1,2,5,6]. However, uncertainty persists regarding the impact of AFCA on the incidence of thromboembolic events [7,8]. While contemporary guidelines generally advocate for continuing oral anticoagulants (OACs) following successful AFCA, the long-term effects of OAC continuation on thrombotic and bleeding complications remain inadequately evaluated in randomized trials [1,2,6]. Earlier observational studies, primarily focusing on warfarin-treated patients, have hinted at a potential reduction in thromboembolic risk with ongoing OAC therapy post-AFCA, albeit with an associated heightened risk of serious bleeding. Observational evidence hints that discontinuing OACs after AFCA may result in a low annual thromboembolic complication rate [[9], [10], [11], [12], [13], [14], [15], [16], [17], [18], [19]]; however, caution is warranted due to study limitations. Further research is needed to guide optimal long-term antithrombotic management post-AF ablation.

The AnticoaguLation ONE year after Ablation of Atrial Fibrillation in Patients with Atrial Fibrillation (ALONE-AF trial; NCT04432220) is a multicenter, randomized controlled study focused on determining the best stroke prevention approach for patients at risk following AF ablation, as indicated by their CHA_2_DS_2_-VASc score [20]. For patients who have not experienced atrial arrhythmia recurrences for at least 12 months after AF ablation, the study hypothesizes that discontinuing OAC therapy will lower the risk of adverse outcomes, such as stroke, systemic embolism, and major bleeding, compared to using direct oral anticoagulants (DOACs).

## 2 Methods

### 2.1 Study design

**Fig. 1:**
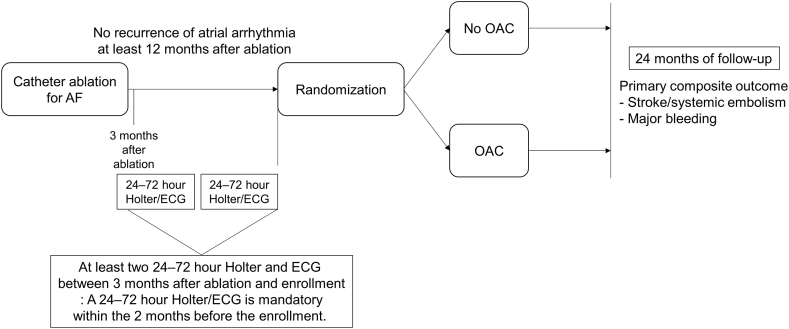
Flow chart of the ALONE-AF trial.

The ALONE-AF trial represents a multicenter, open-label, prospective, randomized superiority trial designed to enroll participants at risk for stroke who have not had any recurrences of atrial arrhythmia for at least 12 months following AF ablation. The study's overall flowchart is depicted in Fig. 1. The study protocol received approval from the institutional review board of each participating center, and patient enrollment commenced in July 2020.

### Funding

The trial is funded by Samjin Pharmaceutical Co Ltd and a grant from the Patient-Centered Clinical Research Coordinating Center (PACEN), funded by the 10.13039/100009647Ministry of Health & Welfare, Republic of Korea (Grant No. HC19C0130).

### 2.2 Study participants and enrollment criteria

All patients with non-valvular AF undergoing their first AFCA procedure will be screened for eligibility. To be eligible for enrollment, patients must meet the following three criteria: 1) age between 19 and 80 years; 2) a CHA_2_DS_2_-VASc score of at least 1 for men or 2 for women; and 3) no atrial arrhythmia recurrence for at least 12 months post-AF ablation, defined as the absence of ≥30 s of atrial fibrillation (AF), atrial flutter (AFL), or atrial tachycardia (AT) in at least two 24–72 h Holter and electrocardiogram (ECG) recordings conducted beyond 3 months after the ablation and before enrollment (requiring at least one 24–72 h Holter monitor and ECG recording within the 2 months prior to enrollment). Patients will be enrolled regardless of the lesion set or energy source used for AF ablation. Key exclusion criteria include: 1) significant renal or liver disease; 2) requirement for anticoagulation for reasons other than AF (such as moderate-to-severe mitral valve stenosis, the presence of a mechanical heart valve, or a history of deep vein thrombosis); and 3) significant structural heart disease. Detailed information on the inclusion and exclusion criteria is presented in Table 1. Written informed consent will be obtained from each subject prior to enrollment.

**Table 1:** Inclusion and exclusion criteria.

Inclusion criteria
1. Age between 19 and 80 years
2. CHA_2_DS_2_-VASc score ≥1 (male) or ≥ 2 (female)
3. No recurrence of atrial arrhythmia at least 12 months after their first-time catheter ablation of atrial fibrillation, defined as an absence of ≥30 s of atrial fibrillation, atrial flutter, or atrial tachycardia in at least two 24–72 h Holter and electrocardiogram recordings conducted beyond 3 months after the ablation and before enrollment (with at least one 24–72 h Holter and electrocardiogram recording mandatory within the 2 months preceding enrollment)
**Exclusion criteria**
1. Significant liver (aspartate transaminase and/or alanine transaminase >3 times the upper limit of normal) or renal disease (serum creatinine ≥3.5 mg/dl or creatinine clearance <30 ml/min)
2. Requiring anticoagulation due to surgery with a mechanical prosthetic valve, moderate-to-severe mitral stenosis, or deep vein thrombosis
3. Significant structural heart disease (moderate-to-severe mitral regurgitation, severe valvular regurgitation or stenosis, dilated cardiomyopathy, or hypertrophic cardiomyopathy)
4. Active malignancy
5. Pregnancy or breast-feeding
6. Life expectancy <1 year
7. Refuse or enable to understand the written informed consent

### 2.3 Randomization and study procedures

Eligible patients will be randomized to receive OAC therapy or no OAC therapy in a 1:1 ratio. Randomization will be conducted using a web-response permuted-block method at each participating center. Participants allocated to the OAC therapy group will receive apixaban at a dose of 5 mg twice daily. The apixaban dose will be reduced to 2.5 mg twice daily for patients who meet at least two of the following conditions: 1) age 80 years or older, 2) body weight 60 kg or less, and 3) serum creatinine level above 1.5 mg/dL [21]. In cases where a subject experiences intolerable adverse effects with apixaban, an alternative DOAC may be prescribed. Subjects assigned to the no OAC therapy group will not receive any oral anticoagulant. However, antiplatelet therapy may be administered if clinically indicated (e.g., for percutaneous coronary intervention or acute coronary syndrome) in both groups. All patients underwent ECG at each visit and 24- to 72-h Holter monitoring at least every six months after enrollment. Additionally, Holter monitoring or event recordings were conducted whenever patients reported symptoms of palpitations suggestive of arrhythmia recurrence. If a patient experiences a documented recurrence of atrial arrhythmia or undergoes repeat AFCA during the study period, they will be censored at that point and prescribed anticoagulation based on the patient's stroke risk or periprocedural needs. Subjects will be followed up for a maximal duration of 24 months.

### 2.4 Study outcomes

The primary outcome, assessed at the 2-year mark following randomization, is a composite endpoint comprising stroke, systemic embolism, and major bleeding (see Table 2). Stroke is defined as a sudden, focal neurologic deficit resulting from a presumed cerebrovascular cause that persists for more than 24 h and is not attributable to a readily identifiable cause, such as a tumor or seizure [22]. Systemic embolism is defined as abrupt vascular insufficiency accompanied by clinical or radiological evidence of arterial occlusion, occurring in the absence of other likely mechanisms (e.g., trauma, atherosclerosis, or instrumentation). Major bleeding events will be defined according to established criteria [22]. Major bleeding was defined by the International Society on Thrombosis and Hemostasis (ISTH) criteria [23].

**Table 2:** Study outcomes.

Primary outcome
A composite of stroke, systemic embolism, and major bleeding (defined by the ISTH criteria)
**Secondary outcome**
Stroke
Systemic embolism
ISTH major bleeding
ISTH clinically relevant non-major bleeding
All-cause death
Myocardial infarction
Pulmonary thromboembolism
Hospitalization
AFQET
K-MoCA
Frailty (Questionnaire and grip strength)

AFQET, Atrial Fibrillation Effect on QualiTy-of-Life; ISTH, International Society on Thrombosis and Hemostasis; K-MoCA, Korean version of Montreal Cognitive Assessment.

Secondary outcomes of the study will include the individual components of the primary outcome, namely stroke, systemic embolism, and major bleeding events. Additionally, we will evaluate the incidence of clinically relevant nonmajor bleeding, as defined by the ISTH criteria [24]. Additional secondary outcomes encompass all-cause mortality, myocardial infarction (MI), pulmonary thromboembolism (PTE), transient ischemic attack (TIA), and hospitalization due to any cause. The definitions of MI and PTE are presented in Supplement. TIA will be defined as the presence of a new focal neurologic deficit presumed to be vascular in origin, with signs or symptoms lasting less than 24 h, without evidence of infarction as assessed by brain imaging [25]. The Atrial Fibrillation Effect on QualiTy-of-life (AFEQT) [26], frailty assessments (including questionnaires and grip strength measurement), and the Korean-Montreal Cognitive Assessment will be evaluated at baseline, as well as at the 1-year and 2-year follow-up time points. Adjudication of study outcomes will be performed by an independent clinical event adjudication committee, which will remain blinded to the primary results of the study.

### 2.5 Sample size estimation

Based on the assumptions provided, including an annual primary outcome rate of 4.6 % in the OAC therapy group (comprising a 1.0 % annual stroke/systemic embolism rate after AFCA and a 3.6 % annual major bleeding rate observed in a pivotal randomized trial of DOAC) [[27], [28], [29]], the total expected primary outcome rate over 2 years was estimated as 9.2 %. This study aims to show that no OAC therapy will achieve a 5.0 % absolute risk reduction over 2 years (equivalent to a 54 % relative risk reduction driven by the reduction of major bleeding) compared with OAC therapy [29,30]. Considering an anticipated 7 % dropout, and aiming for 80 % power and a 5 % two-sided alpha error, a total of 840 patients will be required, with 420 patients allocated to each arm of the trial.

### 2.6 Data collection and management

A baseline evaluation will be carried out to assess the patients' demographics, electrocardiographic data, comorbidities, quality of life, and procedural data related to AFCA. All collected data will be anonymized before being entered into the online database (https://icreat.nih.go.kr). Data collection will occur both at the baseline and after each follow-up evaluation. An independent data monitoring committee will continuously review the study's execution.

### 2.7 Statistical analyses

Continuous variables will be expressed as means ± standard deviations or medians with interquartile ranges, depending on the distribution. Categorical variables will be displayed as frequencies and percentages. For comparisons, the Student's t-test or Mann-Whitney *U* test will be employed for continuous variables, while the chi-square test or Fisher's exact test will be utilized for categorical variables, as suitable.

The primary analyses will be performed on the intention-to-treat (ITT) population. For the primary objective, the cumulative event rate during clinical follow-up will be estimated using the Kaplan-Meier method, with a 95 % confidence interval calculated for the difference in event rates. As a sensitivity analysis, the primary endpoint analysis will also be performed on the per-protocol (PP) population. The ITT population will include all randomized patients, compared according to their assigned group regardless of the treatment actually given. The PP population will exclude patients with protocol deviations, including those found to be ineligible, those without informed consent, or those for whom the randomized therapy was not implemented. For secondary endpoints, the incidence or cumulative incidences of each endpoint will be calculated using Kaplan-Meier plots for comparisons. Missing variables will not be imputed for planned analyses, except where specified otherwise. Patients with missing values will be excluded from variable-related analyses but included in analyses not related to the missing variable. Patients lost to follow-up and subsequently lost to assessment of the primary endpoint will be considered censored in the estimation of Kaplan-Meier event rates There is no planned formal interim analysis or guidelines for stopping the study. However, the data and safety monitoring board (DSMB) will review safety data in a blinded manner, with the DSMB statistician providing unblinded summary tables. The DSMB will discuss and determine whether early termination is required due to safety concerns.

Subgroup analyses will be conducted to compare the hazard ratio (HR) of no OAC therapy against OAC therapy, stratified by prespecified subgroups including age (<65 vs. ≥65 years), sex (male vs. female), diabetes (Yes vs. No), hypertension (Yes vs. No), heart failure (Yes vs. No), vascular disease (Yes vs. No), AF type (paroxysmal vs. non-paroxysmal), and time from diagnosis to ablation (<1 year vs. ≥1 year).

## 3 Discussion

Optimal long-term OAC therapy post-AFCA remains unclear, with varying practices observed worldwide. A survey from Canada indicates that more than 95 % of electrophysiologists would consider stopping OAC therapy if their patients have a CHADS_2_ score of 1 or lower and no recurrences of atrial arrhythmia [31]. In contrast, an observational study conducted in Japan demonstrates that over half of the patients continued OAC therapy at 1 year post-ablation. The study reveals a higher rate of OAC therapy continuation in patients with a higher CHADS_2_ score (at least 3) compared to those with a CHADS_2_ score of 2 or less [19]. Consistently, a survey of European electrophysiologists shows that 16 % would be comfortable discontinuing OAC therapy after AFCA, even though their patients have a high stroke risk as indicated by a CHADS_2_ score of at least 2 [32].

Noseworthy et al. shows that OAC discontinuation in the long term after ablation is associated with an increased risk of cardioembolism for high-risk patients [33]. Kanaoka et al. demonstrates that the thromboembolic risk in patients undergoing AFCA is lower compared to the general AF population not receiving OACs [19]. Moreover, discontinuing OAC treatment, even in patients with a CHADS_2_ score of 2, does not lead to an increased incidence of thromboembolism but is associated with a decreased risk of major bleeding. Therefore, the routine continuation of OAC therapy in patients with intermediate stroke risk may not be advisable. Given the acknowledged lack of evidence supporting current guideline recommendations on this topic, there is equipoise, justifying the need for a randomized trial to address this question.

In summary, the ALONE-AF trial seeks to address this gap by investigating whether OAC discontinuation enhances the net clinical benefit among patients who have undergone successful AF ablation. The trial's findings are expected to provide valuable insights into the optimal antithrombotic regimen for post-ablation patients with stroke risk factors.

## 4 Ethics statement

The study protocol received approval from the institutional review board of each participating center, and patient enrollment began in July 2020. The trial is registered on ClinicalTrials.gov on June 12, 2020 (NCT04432220). All participants will provide informed consent to participate in the study.

## Funding

This research received support from Samjin Pharmaceutical Co Ltd and a grant from the Patient-Centered Clinical Research Coordinating Center (PACEN), funded by the 10.13039/100009647Ministry of Health & Welfare, Republic of Korea (Grant No. HC19C0130).

## Data availability statement

No data is used because this manuscript is a study protocol. Data associated with this trial will be made available from the corresponding author on request, considering privacy-sensitive information.

## CRediT authorship contribution statement

**Daehoon Kim:** Writing – review & editing, Writing – original draft, Investigation, Conceptualization. **Jaemin Shim:** Investigation, Data curation. **Eue-Keun Choi:** Supervision, Data curation. **Il-Young Oh:** Supervision, Data curation. **Jun Kim:** Supervision, Data curation. **Young Soo Lee:** Supervision, Data curation. **Junbeom Park:** Supervision, Data curation. **Jum-Suk Ko:** Supervision, Data curation. **Kyoung-Min Park:** Supervision, Data curation. **Jung-Hoon Sung:** Supervision, Methodology. **Hyung Wook Park:** Supervision, Data curation, Conceptualization. **Hyung-Seob Park:** Supervision, Data curation. **Jong-Youn Kim:** Supervision, Project administration. **Hee Tae Yu:** Supervision, Project administration, Methodology, Investigation, Formal analysis, Data curation. **Tae-Hoon Kim:** Validation, Supervision, Resources, Investigation, Data curation, Conceptualization. **Boyoung Joung:** Writing – review & editing, Visualization, Validation, Supervision, Resources, Project administration, Funding acquisition.

## Declaration of competing interest

The authors declare the following financial interests/personal relationships which may be considered as potential competing interests:Boyoung Joung reports financial support was provided by Korea Ministry of Health and Welfare. Boyoung Joung reports financial support was provided by Samjin Pharm Co Ltd. Boyoung Joung has served as a speaker for 10.13039/100004326Bayer, BMS/10.13039/100004319Pfizer, Medtronic, and 10.13039/501100002973Daiichi-Sankyo and received research funds from Samjin, 10.13039/100019265Yuhan, Medtronic, Boston Scientifics and 10.13039/100008977Abbott Korea. No fees were received personally. The remaining authors have no conflicts of interest to declare. If there are other authors, they declare that they have no known competing financial interests or personal relationships that could have appeared to influence the work reported in this paper.
