# Supplementary material for: The anticoagulation one year after ablation of atrial fibrillation in patients with atrial fibrillation (ALONE-AF) trial: Study protocol
Source: Heliyon. 2024 Aug 16;10(16):e36506. doi: 10.1016/j.heliyon.2024.e36506 (PMC11379993; doi:10.1016/j.heliyon.2024.e36506)
Supplement: Multimedia component 1 [file mmc1.docx]

**Definition of Myocardial Infarction**

Myocardial Infarction will be considered to have occurred if any of the following criteria are

met:

- Rise and/or fall of cardiac biomarker values (preferably cardiac troponin) with at least one value above the 99th percentile upper reference limit (URL) and with at least one of the following:
  - Symptoms of ischemia
  - New or presumed new significant ST-segment – T wave changes or new left bundle branch block (LBBB)
  - Development of pathological Q waves in the ECG
  - Imaging evidence of a new loss of viable myocardium or new regional wall motion abnormality
  - Identification of an intracoronary thrombus by angiography or autopsy

OR

- Cardiac death with symptoms suggestive of myocardial ischemia and presumed new ischemic ECG changes or new LBBB, but death occurred before cardiac biomarkers were obtained, or before cardiac biomarker values would be increased.

OR

- Percutaneous coronary intervention (PCI) related MI is arbitrarily defined by elevation of cardiac troponin values (>5 x 99th percentile URL) in patients with normal baseline values (≤ 99th percentile URL) or a rise > 20% if the baseline values are elevated and are stable or falling. In addition, at least one of the following:
  - Symptoms suggestive of myocardial ischemia
  - New ischemic ECG changes
  - Angiographic findings consistent with a procedural complication
  - Imaging demonstration of new loss of viable myocardium or new regional wall motion abnormality

OR

- Stent thrombosis associated with MI when detected by coronary angiography or autopsy in the setting of myocardial ischemia and with a rise and/or fall of cardiac biomarker values with at least one value above the 99th percentile URL

OR

- Coronary artery bypass grafting (CABG) related MI is arbitrarily defined by elevation of cardiac biomarker values (> 10 x 99th percentile URL) in patients with normal baseline values (≤ 99th percentile URL). In addition, at least one of the following:
  - New pathological Q waves or new LBBB
  - Angiographic documented new graft or new native coronary artery occlusion
  - Imaging evidence of new loss of viable myocardium or new regional wall motion abnormality

**Definition of pulmonary thromboembolism**

The diagnosis of pulmonary thromboembolism requires one or more among:

- An intraluminal filling defect at CT pulmonary angiography
- An intraluminal filling defect, or a new sudden cut-off of vessels more than 2.5mm in diameter at pulmonary angiogram
- A perfusion defect of at least 75% of a segment with a local normal ventilation result (high probability) on ventilation/perfusion lung scan (VQ scan)
- In unsuspected pulmonary thromboembolism, there must be one or more filling defect in segmental or more proximal arteries at chest CT pulmonary angiography
